# Genotype-phenotype correlation in 75 patients with small supernumerary marker chromosomes

**DOI:** 10.1186/s13039-020-00494-2

**Published:** 2020-07-14

**Authors:** Tingting Li, Haiquan Sang, Guoming Chu, Yuanyuan Zhang, Manlong Qi, Xiaoliang Liu, Wanting Cui, Yanyan Zhao

**Affiliations:** 1 Department of Clinical Genetics, Shengjing Hospital of China Medical University, 36 Sanhao Street, Heping District, Shenyang, 110003 China; 2 Department of General Surgery, the Fourth Affiliated Hospital of China Medical University, Shenyang, China

**Keywords:** Small supernumerary marker chromosomes, Next-generation sequencing, Prenatal diagnosis, Genetic counseling

## Abstract

**Background:**

Small supernumerary marker chromosomes (sSMCs) are rare structural abnormalities in the population; however, they are frequently found in children or fetuses with hypoevolutism and infertile adults. sSMCs are usually observed first by karyotyping, and further analysis of their molecular origin is important in clinical practice. Next-generation sequencing (NGS) combined with Sanger sequencing helps to identify the chromosomal origins of sSMCs and correlate certain sSMCs with a specific clinical picture.

**Results:**

Karyotyping identified 75 sSMCs in 74,266 samples (0.1% incidence). The chromosomal origins of 27 of these sSMCs were detected by sequencing-related techniques (NGS, MLPA and STR). Eight of these sSMCs are being reported for the first time. sSMCs mainly derived from chromosomal X, Y, 15, and 18, and some sSMC chromosomal origins could be correlated with clinical phenotypes. However, the chromosomal origins of the remaining 48 sSMC cases are unknown. Thus, we will develop a set of economical and efficient methods for clinical sSMC diagnosis.

**Conclusions:**

This study details the comprehensive characterization of 27 sSMCs. Eight of these sSMCs are being reported here for the first time, providing additional information to sSMC research. Identifying sSMCs may reveal genotype-phenotype correlations and integrate genomic data into clinical care.

## Background

Small supernumerary marker chromosomes (sSMCs) are structural abnormalities whose origins cannot be characterized by conventional cytogenetics alone but require molecular approaches. It is known that 70% of sSMCs are de novo, 20% are inherited from the mother, and 10% come from the father [1]. sSMCs are often derived from maternal meiosis I/II errors, trisomic/monosomic rescue, or fertilization errors [2, 3]. sSMCs are equal to or smaller than chromosome 20 in size and often have abnormal morphology (e.g., inverted duplication, minute, or ring). Many of them are derived from the short arms or pericentromeric regions of chromosomes. Nearly 70% of sSMC carriers are clinically normal; however, 30% are abnormal. Patients carrying sSMCs have developmental delays, intellectual disabilities, mixed gonadal dysgenesis (MGS), or infertility, depending on the origin of the sSMC. The treatment of these patients was based on different symptoms until the molecular characterization of sSMCs was developed.

In this study, we identified 75 sSMC cases in 74,266 patients seen in our department from 2015 to 2018 by karyotyping. Fifty-seven of the cases were subjected to molecular analysis, and the remaining 18 were not characterized further. Next-generation sequencing (NGS) is a fast high-output sequencing technique used to determine copy number variations [4]. We combined NGS, multiplex ligation-dependent probe amplification (MLPA), and short tandem repeat (STR) analysis to identify the origins of the sSMCs in our study. The molecular components of 27 of the sSMCs were identified. Thirty of the sSMCs subjected to molecular analysis did not have any pathogenic information in original chromosomal.

sSMCs were first detected by conventional cytogenetic banding analysis, which is weak for identifying their molecular component. This study aimed to identify the origins of sSMCs diagnosed in our department over the last 4 years. This application may help recognize syndromes from which sSMC patients suffer, establish suitable and specific therapy, or even predict syndromes that will develop in the future. Such an application will be of great value in clinical genetic diagnosis and genetic counseling.

## Results

### Distribution of cases

**Fig. 1:**
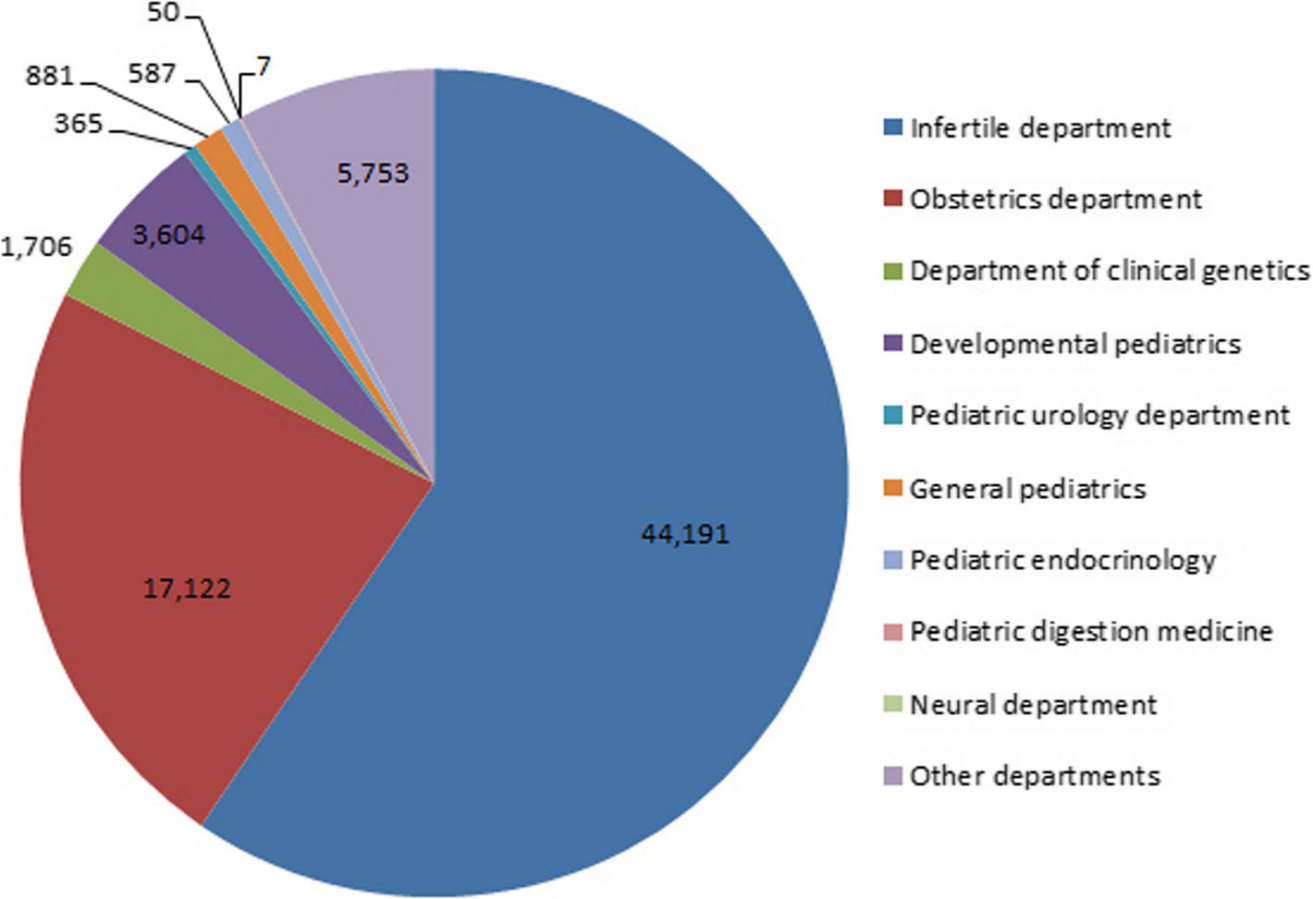
Departmental distribution of patients in the last 4 years. The patients in this study came mainly from the infertility (44,191), obstetrics (17,122), and developmental pediatric (3,604) departments of Shengjing hospital

A total of 74,266 samples were analyzed for genetic diagnosis from the infertility, pediatrics, and obstetrics departments of Shengjing hospital (Fig. 1). In particular, we studied 75 sSMC carriers (0.1% in total), including 23 adults with infertility or habitual abortion (23/75, 30.67%), 20 children with severe developed delay, MGS or gynandromorphism (20/75, 26.67%), 23 fetuses with intrauterine growth retardation or abnormal ultrasonic structures (23/75, 30.67%), and nine unsyndromatic sSMC cases (9/75, 12%). We performed NGS, MLPA, and STR on 57 sSMCs and identified the chromosomal origins for 27 of these cases (Table 1). The chromosomal origins of the remaining 48 cases are still unknown (Table 2). These data suggested that most sSMC cases have clinical syndromes, which might be correlated with their clinical phenotypes.

**Table 1:** The information of 27 identified sSMC patients

Patient NO.	Gender/age at diagnosis	Studied material	Cytogenetics	Final result of the sSMC	Test methods and results	Clinical symptoms	Age of gravida/karyotypes of parents	De novo/ inherited
61166^a^	male/14 m	PBL	45,X[2]/46,X,+mar[15]	del(Y)(pter→q11.222::q11.223→qter), first report	NGS:del(Y)(p11.2)×0.5 (2.7 Mb), del(Y)(q11.222→q11.223)×0 (2.2 Mb). AZF b, −d and -c regions: deleted. STR:AMEL (Xp22.2:Yp11.2): 2:1. SRY (Yp11.31): positive.	Hypospadias, right cryptorchidism,term birth (BW 2.15 kg). He performed the corrective surgery before karyotyping report.	n.a.	n.a.
W02938	male/13 m	PBL	45,X[26]/46,X,+mar[19]	min(Y) with SRY	STR:AMEL(Xp22.2:Yp11.2): 2:1. SRY (Yp11.31): positive. DYS448(Yq11.223): negative.	Hypospadia, congenital testicular hypoplasia. His small penis was bent towards the abdomen side, and showed phimosis.	n.a.	n.a.
69433	female/6y	PBL	45,X[13]/46,X,+mar[8]	min(Y)	MLPA: Y was abnormal.	Pygmyism, asitia. H:106 cm, W:17.2 kg, BW:2.9 kg.	n.a.	n.a.
61680	male/29y	PBL	46,X,mar[9]/46,XY[9]	min(Y) with SRY	AZF-d and -c regions: deleted. STR:AMEL (Xp22.2:Yp11.2): 1:1. SRY (Yp11.31): positive.	Azoospermatism	n.a.	n.a.
62091	male/31y	PBL	45,X[8]/46,X,mar[7]	min(Y) with SRY	AZF-b, −d and -c regions: deleted. STR:AMEL (Xp22.2:Yp11.2): 1:1. SRY (Yp11.31): positive.	Azoospermatism	n.a.	n.a.
77297	male/26y	PBL	46,X,+mar	min(Y) with SRY	AZF all regions: deleted. STR:AMEL (Xp22.2:Yp11.2): 1:1. SRY (Yp11.31): positive.	Azoospermatism	n.a.	n.a.
80794	male/32y	PBL	46,X,+mar1[4]/46,X,+mar2[7]/47,X,+mar3,+mar4[12]	min(Y) with SRY	AZF-b,-d and -c regions: deleted. STR:AMEL (Xp22.2:Yp11.2): 1:1. SRY (Yp11.31): positive.	Azoospermatism, infertile	n.a.	n.a.
98139	male/28y	PBL	46,X,+mar?	min(Y) with SRY	AZF all regions: deleted. STR:AMEL (Xp22.2:Yp11.2): 1:1. SRY (Yp11.31): positive.	Infertile, azoospermatism.	n.a.	n.a.
W01824	male/31y	PBL	45,X [15]/46,X,+mar[10]	min(Y) without SRY	AZF all regions: deleted. STR:AMEL (Xp22.2:Yp11.2): 2:1. SRY (Yp11.31): negative.	Infertile, azoospermatism. He had undergone remedial surgery for hypospadias and cryptorchidism when he was 5 years old. Magnetic resonance imaging (MRI) showed right spermatophore hypogenesis, and left spermatophore containing a mass.	n.a.	n.a.
150677	n.a./prenatal	AF	45,X[1]/46,X,+mar[19]	min(Y) with SRY	STR:AMEL (Xp22.2:Yp11.2): 1:1. SRY (Yp11.31): positive. DYS448(Yq11.223): positive.	NIPT indicated abnormal heterosomes. Gravida was G4P1,and had nature labour twice. Spousal AZF regions was normal.	38/46,XX;46,XY	de novo
162047	n.a./prenatal	AF	46,X,+mar(Y?)	min(Y) with SRY	STR:AMEL (Xp22.2:Yp11.2): 1:2. SRY (Yp11.31): positive. DYS448(Yq11.223): negative.	NIPT indicated abnormal heterosome. Gravida was G2P1.	33/n.a.	n.a.
171276^a^	n.a./prenatal	AF	45,X[2]/47,X,+mar1,+mar2[1]/46,X,+mar1[47]	mar1: min(Y)(:p11.31→qter), mar2: inv dul(Y)(q11.221→p11.31::p11.31→q11.221), first report	NGS: dup(Y)(p11.31→q11.221)×3, del(Y)(q11.221→q12)×1, mosaic 45,X. STR: AMEL(X:Y): 1:2. SRY (Yp11.31): positive. DYS448(Yq11.223): negative.	NT: 4.7 mm(> 3.0 mm). Gravida underwent NGS in another hospital.	24/46,XX.	n.a.
69813	male/6y	PBL	47,XY,+mar	inv dup(15)(q11.2 ~ 13.3), dul(15)q(13.3)	NGS: dup(15)(q11.2→q13.3)×4 (8.2 Mb), dup(15)q(13.3)×3 (1.6 Mb)	Hypoevolutism, hypophrenia, epilepsy. He could only say a few words. His EEG demonstrated epilepsy changes.	n.a.	n.a.
W03987	male/31y	PBL	47,XY,+mar	inv dup(15)(q11.2)	NGS: polymorphism dul(15)(q11.2)(22740001–23520000)×4 (0.78 Mb). AZF: normal. SRY: positive.	Infertile, asthenospermia.	n.a.	n.a.
W04210	female/25y	PBL	47,XX,+mar	min(15)(:q11.2→q13.1:)	NGS: dup(15)(q11.2→q13.1)×3 (5.64 Mb)	Hyperspasmia. She had hyperspasmia for twenty years. Her hyperspasmia occurred during sleep, with tongue biting, foaming at the mouth, and gatism, looking like epilepsy.	n.a.	n.a.
70532	male/2y	PBL	47,XY,+mar	inv dup(15)(q11.2)	MLPA: 3 points (two of SNRPN and one of UBE3A) of 15q11.2 were a heterozygous duplicated mutation.	Autism	n.a.	n.a.
83411	female/5y	PBL	47,XX,+mar	inv dup(15)(q11.2)	MLPA: 3 points of 15q11.2 were heterozygous duplicated mutation.	Hypoevolutism and mental retardation. She could not sit on her own at 1 year old and could not walk at 3 years old. MRI showed that her left lobus frontalis was partly demyelinated. Ultrasound of the heart revealed a ventricular septal defect, left to right ventricle shunt, wide coronary sinus, and persistent left superior vena cava.	n.a.	n.a.
96862	female/18 m	PBL	47,XX,+mar	inv dup(15)(q11.2)	MLPA: 3 points of 15q11.2 were heterozygous duplicated mutation.	Hypoevolutism. She could not walk steadily or pick up things with her fingers, and had poor communication. MRI of the cerebrum showed that both sides of the hemisphere were not full.	n.a.	n.a.
92568^a^	female/12y	PBL	45,X[7]/46,X,+mar[13]	r(X)(::p11.23→q21.1::), first report	NGS: 45,X[57%]/46,X,r(X)(p11.23→q21.1)[43%]	She was suspected Turner syndrome, and injected GH for 1 year.	n.a.	n.a.
W09834^a^	female/14 m	PBL	45,X[4]/46,X,+mar[26]	min(X)(:p11.2→q13.2:), first report	NGS: partly 45,X: X (pter→p11.21) x1, X(q13.2→qter)×1. SRY: negative.	Turner syndrome.	n.a.	n.a.
61259	male/57d	PBL	47,XY,+mar	inv dup(18)(pter→p11.21::p11.21→pter)	NGS: dup(18)(p11.32→p11.21)×4 (15.3 Mb)	Neonatal feeding problem, pneumonia. He had microcephaly, low-set ears and often gazed look.	n.a.	n.a.
172168	female/prenatal	AF	47,XX,+mar	inv dup(18)(pter→p11.21::p11.21→pter)	NGS: dup(18)(p11.32→p11.21)×4. STR: normal.	NIPT: the high risk of 18-trisomy syndrome (Edwards syndrome).	38/46,XX.	n.a.
96932^a^	female/4y	PBL	45,X(21ps+) [14]/46,X,+mar,(21ps+)[6]	min(X), min(Y), first report	NGS: 45,X[65%]/46,XY[17%]/46,XX[18%]	Hypoevolutism. She grew slowly after birth, with W: 12.5 kg, H: 93 cm, (H/A ≤ 2SD). She had skin rash on the face, webbed neck, and short stature, looking like Turner syndrome. Her bone age was 3.5 years old, and 4 left carpals were sclerotized. Ultrasound showed vestige uterus and no ovary.	n.a.	n.a.
172990^a^	female/prenatal	AF	47,XX,+mar	min(9)(pter→p13.1:), first report	NGS: dup(9)(p24.3→p13.1)×3. STR: normal.	NIPT indicated abnormal chrosome 9.	37/46,XX.	n.a.
70963^a^	female/8y	PBL	47,XX,+mar(1qh+) [18]/46,XX(1qh+)[12]	min(20)(:p12.3→q11.22:), first report	NGS: mosaic duplication (20)(p12.3→q11.22)×3 (20.1 Mb)	Pygmyism,asitia. She had asitia and was sickly; W: 21.7 kg, H: 115.5 cm, H/A ≤ -2SD. Her 7 left carpals were sclerotized. Her mother’s height was 158 cm and father’s 178 cm. NGS was done at another hospital.	n.a.	n.a.
160246^a^	female/prenatal	AF	160246: 47,XX,+mar	min(11)(:q23.3→qter), first report	NGS: dup(11)(q23.3→q25)×3. STR: normal.	In 2016, her mother got pregnant (numbered 160246). Ultrasound showed that there was a fluid sonolucent area in the nuchal region of 160246. NGS performed at another hospital. In 2017, her mother got pregnant again (numbered 173026). The fetus carried the same balanced translocation, and his NGS results were normal.	29/46,XX,t(11;22)(q23;q12)46,XY	de novo
184290	male/prenatal	AF	47,XY,+mar	inv dup(22)(q11.1 ~ 11.21)	NGS:dup(22)(q11.21)×3(2.46 Mb), dup(22)(q11.1→q11.21)×4. STR: normal.	NT: 3.1 mm. Gravida aborted a fetus with congenital heart disease in 2017.	32/46,XX;46,XY	de novo

^a^The sSMC was reported for the first time

*Abbreviations*: *PBL* peripheral blood, *AF* amniotic fluid, *y* year, *m* month, *d* day, *n.a* not available, *NIPT* non-invasive prenatal testing, *NT* nuchal translucency

**Table 2:** The information of 48 unidentified sSMC patients

Patient NO.	Gender/ age at diagnosis	Studied material	Cytogenetics	Test methods and results	Clinical symptoms	Age of gravida/karyotypes of parents	De novo/ inherited
150234	male/prenatal	AF	47,XX,+mar[23]/46,XX[21]	STR: normal	Diabetes of type II	36/n.a.	n.a.
150693	female/prenatal	AF	48,XX,+18,+mar	STR: 18-trisomy syndrome (Edwards syndrome)	Down’s syndrome screening: high-risk. Advanced maternal age.	43/n.a.	n.a.
151434	male/prenatal	AF	47,XY,+mar	STR: normal. SRY: positive	Ultrasound: ventricular septal defect, small kidney.	31/46,XX;46,XY	de novo
153225	female/prenatal	AF	47,XX,+mar[5]/46,XX[39]	STR: normal	Ambryo develop delay	28/n.a.	n.a.
161045	n.a./prenatal	AF	45,X [11]/46,X,+mar[21]	STR: 45,X	NIPT: abnormal heterosome, NT:2.9 mm	36/n.a.	n.a.
163110	male/prenatal	AF	47,XY,+mar	STR: normal. SRY: positive	Cerebromedullary tube anisotrophy.	30/46,XX.	n.a.
170574	n.a./prenatal	AF	45,X[30]/46,X,+mar[3]	STR: 45,X	Single umbilical artery (SUA), seroperitoneum of fetus	30/n.a.	n.a.
172376	n.a./prenatal	AF	46,X,+mar[17]/45,X[12]	STR: 45,X	NT > 3 mm	33/46,XX.	n.a.
173060	female/prenatal	AF	47,XX,+mar	STR: normal	Down’s syndrome screening: high-risk	26/n.a.	n.a.
180036	female/prenatal	AF	47,XX,+mar[1](SC)/46,XX[35]	STR: normal	Oligohydramnios.	30/n.a.	n.a.
180748	female/prenatal	AF	47,XX,+mar[3](MC)/46,XX[22]	STR: normal	Twins	28/n.a.	n.a.
181010	female/prenatal	AF	47,XX,+mar[1]/46,XX[29]	NIPT: low risk. STR: normal	Ventricular septal defect	37/n.a.	n.a.
183584	male/prenatal	AF	47,XY,+mar[1]/46,XY[29]	STR: normal	Down’s syndrome screening: high-risk(1/346).	31/n.a.	n.a.
184082	female/prenatal	AF	47,XX,+mar[1]/46,XX[29]	STR: normal	Down’s syndrome screening: high-risk	26/n.a.	n.a.
184172	male/prenatal	AF	47,XY,+mar[1](SC)/46,XY[24]	NGS: dup(11)(p15.3→p15.3)×3, dup(6)(p12.32)(32400000–32780000)×3	NT: 2.5 mm	27/n.a.	n.a.
A1045	female/prenatal	UCB	47,XX,+mar		Develop delay for one month.	30/n.a.	n.a.
61200	male/32y	PBL	47,XY,+mar[6]/36,XY[13]	AZF: normal. SRY: positive	Infertile.	n.a.	n.a.
61397	male/24y	PBL	47,XY,+mar	AZF: normal. SRY: positive	Azoospermatism, hyperprolactinemia.	n.a.	n.a.
62254	female/3y	PBL	47,XX,+mar	MLPA: normal	Global developdelay	n.a.	n.a.
63001	male/29y	PBL	47,XY,+mar	AZF: normal. SRY: positive	Asthenospermia	n.a.	n.a.
63411	female/22y	PBL	47,XX,+mar		The mother of a patient with develop delay .	n.a.	n.a.
65676	female/3y	PBL	48,XX,+ 21,+mar[13]/47,XX,+ 21[7]		Heart malformation	n.a.	n.a.
67979	female/9y	PBL	46,X,+mar[14]/45,X[11]		Runtishness	n.a.	n.a.
69235	female/12 m	PBL	46,X,+mar[8]/45,X[12]	MLPA: X was abnormal	Developdelay	n.a.	n.a.
72699	male/3y	PBL	48,XY,+mar1,+mar2	MLPA: normal	Autism.	n.a.	n.a.
73431	male/10y	PBL	48,XY,+mar1,+mar2	SRY: positive	Astigmia and hypometropia.	n.a.	n.a.
73940	female/59y	PBL	47,XX,+mar		n.a.	n.a.	n.a.
7300	male/33y	PBL	47,XY,+mar	NGS: No obvious abnormal was detected. AZF: normal. SRY: positive.	Asthenospermia,teratospermia.	n.a.	n.a.
80039	male/33y	PBL	47,XY,+mar	NGS: A 0.46 Mb section deleted in 6q12, no pathopoiesia information.	Infertile,asthenospermia.	n.a.	n.a.
81882	female/15y	PBL	46,X,mar[11]/45,X[9]		Primary amenorrhea	n.a.	n.a.
85773	male/32y	PBL	47,XY,+mar	NGS: No obvious abnormal was detected.	Infertile, azoospermatism.	n.a.	n.a.
90074	female/9 m	PBL	45,X [8]/46,X,+mar[19]	NGS: 45,X	Hypoevolutism	n.a.	n.a.
91473	female/20y	PBL	46,X,+mar[11]/45,X[10]	SRY: negtive	Primary amenorrhea.Vestigial uterus.	n.a.	n.a.
92243	female/34y	PBL	47,XX,+mar[19]/46,XX[13]	NGS: No obvious abnormal was detected.	Infertile	n.a.	n.a.
92638	female/25y	PBL	45,X,+mar[1]/46,XX [16]/47,XX,+mar[3]		Infertile	n.a.	n.a.
93162	n.a./9 m	PBL	45,X [11]/46,X,+mar [9]		Gynandromorphism	n.a.	n.a.
96704	female/2y	PBL	45,X [17]/46,X,+mar[13]		Pygmyism	n.a.	n.a.
97858	male/30y	PBL	47,XY,+mar	AZF: normal. SRY: positive	Infertile, azoospermatism.	n.a.	n.a.
W00311	female/30y	PBL	47,XX,+mar	NGS: A 0.14 Mb section deleted in 2q32.1, no pathopoiesia information.	G1P0 embryonic stop develop at 11 weeks.	n.a./47,XX, + mar	maternal
W00880	female/53y	PBL	47,XX,+mar		W00311’s mother	n.a.	n.a.
W02523	female/21y	PBL	47,XX,+mar[2]/46,XX[23]		G4P0. Habitual abortion, arrested embryo.	n.a.	n.a.
W03572	female/7 m	PBL	47,XX,+mar[1]/46,XX[29]		Develop delay.	n.a.	n.a.
W06115	female/30y	PBL	47,XX,+mar		G3P1. Arrested embryo twice.	n.a.	n.a.
W06490	female/29y	PBL	47,XX,+mar[26]/46,XX[12]		G2P0. Arrested embryo twice.	n.a.	n.a.
W07384	male/30y	PBL	47,XY,+mar[3]/46,XY[36]		Spouse had one time hydatidiform mole.	n.a.	n.a.
W13749	female/4y	PBL	47,XX,+mar		Developmental retardation.	n.a.	n.a.
W13804	male/18 m	PBL	45,X[12]/46,X,+mar[18]		Hypospadia	n.a.	n.a.
W14357	female/28y	PBL	47,XX,+mar		Pregnant preparation	n.a.	n.a.

*Abbreviations*: *PBL* peripheral blood, *AF* amniotic fluid, *UCB* umbilical cord blood, *y* year, *m* month, *d* day, *n.a* not available, *NIPT* non-invasive prenatal testing, *NT* nuchal translucency

### sSMCs from chromosome Y

**Fig. 2:**
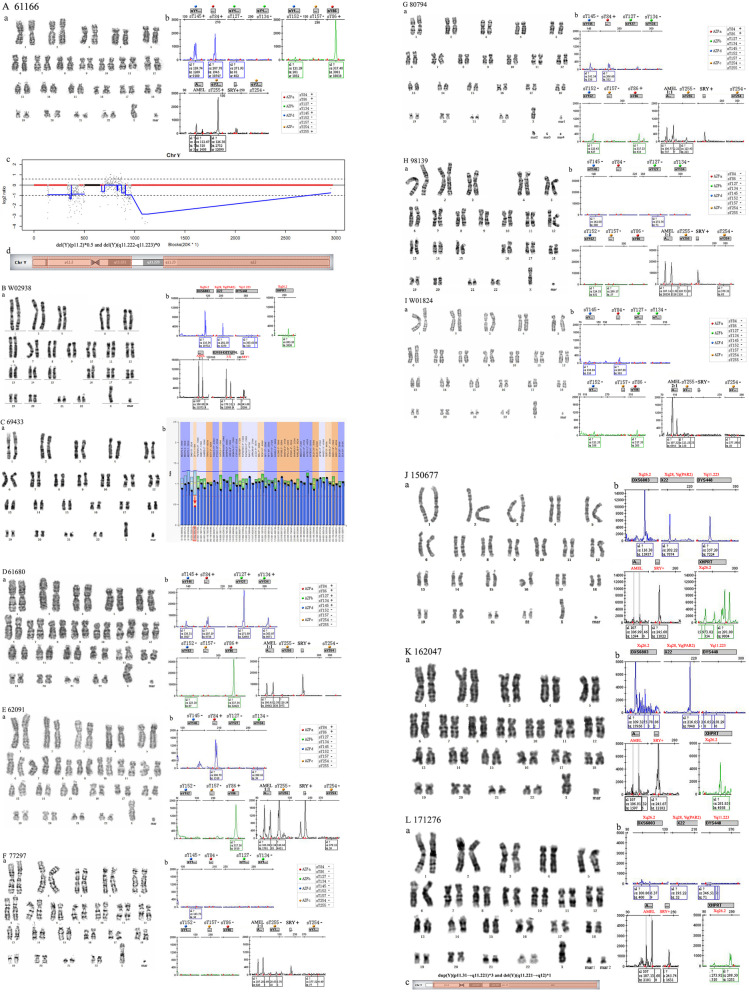
Cytogenetic and molecular results for patients carrying sSMCs derived from min(Y). (**A**) 61166: (**a**) The karyotype was detected by G-banding. (**b**) AZF-b, AZF-d, and AZF-c regions were deleted. The SRY was positive. (**c**) NGS identified two deletions on chromosome Y. (**d**) The location of the sSMC on chromosome Y is highlighted in red. (**B**) W02938: (**a**) The karyotype was mosaic. (**b**) STR AMEL(X:Y) was 2:1, DYS448(Yq11.223) was not detected (negative), but SRY was positive. (**C**) 69433: (**a**) The karyotype was mosaic. (**b**) MLPA detected an abnormal Y in “Y-002.889246”. The data for patients 61680, 62091, 77297, 80794, 98139 and W01824 were presented separately from (**D**) to (**I**). (**a**) The karyotypes were revealed by G-banding. (**b**) STR analysis detected deletions in the AZF regions. The date of 150677, 162047 and 171276 were presented separately from (**J**) to (**L**). (**a**) The karyotypes were revealed by G-banding. (**b**) STR detected X and Y chromosomes. (**L**) 171276: (**c**) The location of the sSMC on chromosome Y is highlighted in red

Twelve sSMCs were derived from chromosome Y. Patients 61166 and W02938 were sexually abnormal boys, showing similar characteristics to Turner syndrome with androgynous. Results showed the sSMCs were derived from a minute Y chromosome with SRY (Fig. 2A, B). Patient 69433 grew up as a girl. The MLPA analysis indicated that the sSMC was derived from min(Y) (Fig. 2C). Patients 61680, 62091, 77297, 80794, 98139 and W01824 were adult men with azoospermia and infertility. STR analysis showed that their sSMCs came from min(Y) (Fig. 2D-I, Table 3). Samples 150677, 162047, and 171276 were from amniotic fluid. The STR analysis results demonstrated that the sSMCs were from min(Y) (Fig. 2J-L).

**Table 3:** The results of AZF

Regions		61166	61680	62091	77297	80794	98139	W01824
AZFa	sY84	+	+	+	–	+	–	–
	sY86	+	+	+	–	+	–	–
AZFb	sY127	–	+	–	–	–	–	–
	sY134	–	+	–	–	–	–	–
AZFd	sY145	+	+	–	–	–	–	–
	sY152	–	–	–	–	–	–	–
AZFc	sY157	–	–	–	–	–	–	–
	sY254	–	–	–	–	–	–	–
	sY255	+	–	–	–	–	–	–
SRY		+	+	+	+	+	+	–

### sSMCs from chromosome 15

**Fig. 3:**
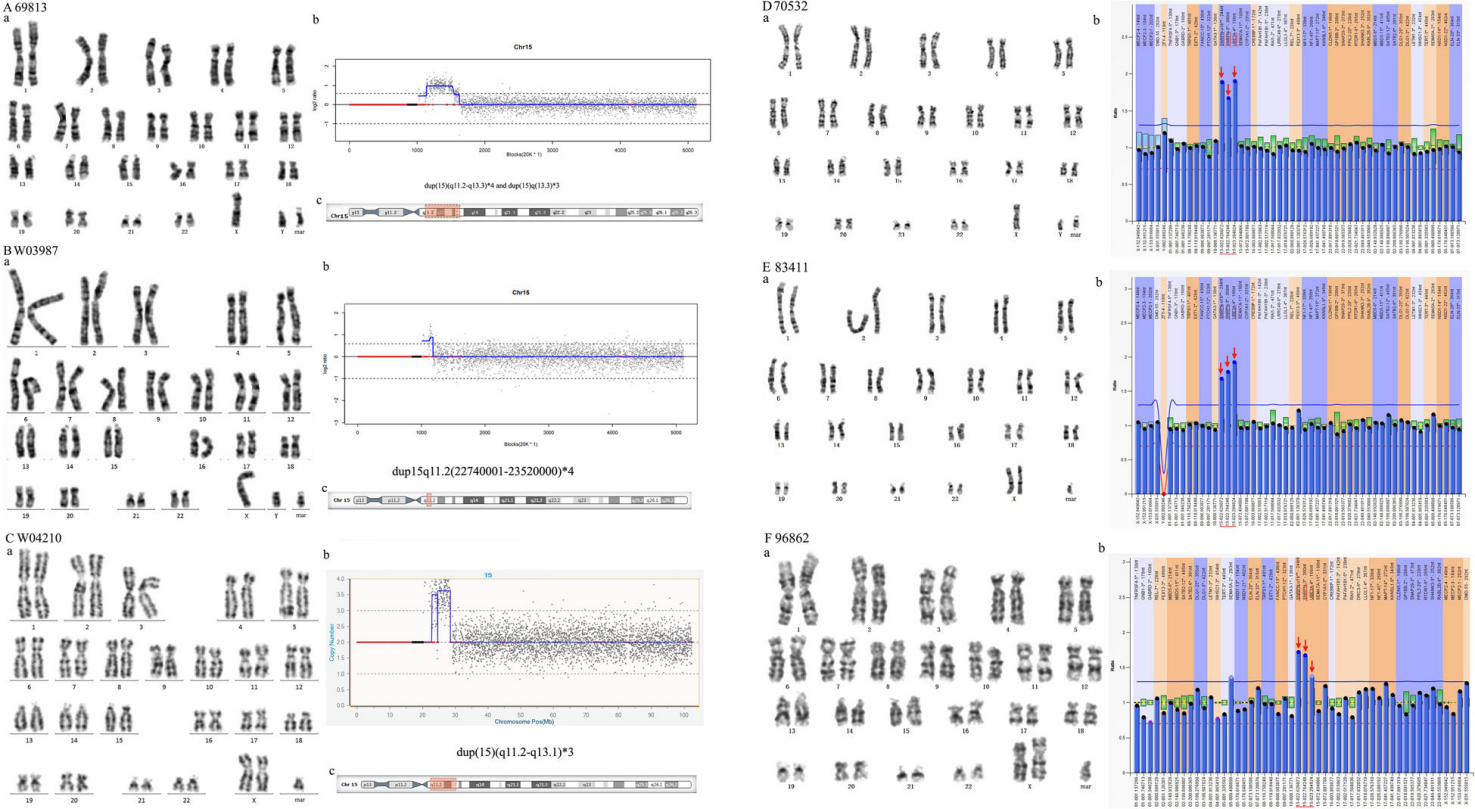
Cytogenetic and molecular results for patients carrying sSMCs derived from inv dul(15) and min(15). The date of 69813, W03987 and W04210 were presented separately from (**A**) to (**C**). (**a**) The karyotypes were revealed by G-banding. (**b**) NGS identified duplications on chromosome 15. (**c**) The location of the sSMC on chromosome15 is highlighted in red. The date of 70532, 83411 and 96862 were presented separately from (**D**) to (**F**). (**a**) The karyotypes were revealed by G-banding. (**b**) The MLPA revealed heterozygous duplicated mutations at 15q11.2

The sSMCs of six patients were derived from chromosome 15. NGS identified duplications on chromosome 15 for patients 69813 and W03987 (Fig. 3A, B). MLPA revealed that patients 70532, 83411, and 96862 had a heterozygous duplicated mutation at 15q11.2 (Fig. 3D-F). These five patients carried sSMCs derived from inv dul(15). The sSMC of patient W04210 was from min(15) (Fig. 3C). Five of these cases showed clinical features of Dup15q syndrome (e.g., hypoevolutism or autism). In contrast, case W03987 with inv dup(15)(q11.2) was polymorphic without the features of Dup15q syndrome.

### sSMCs from chromosome X

**Fig. 4:**
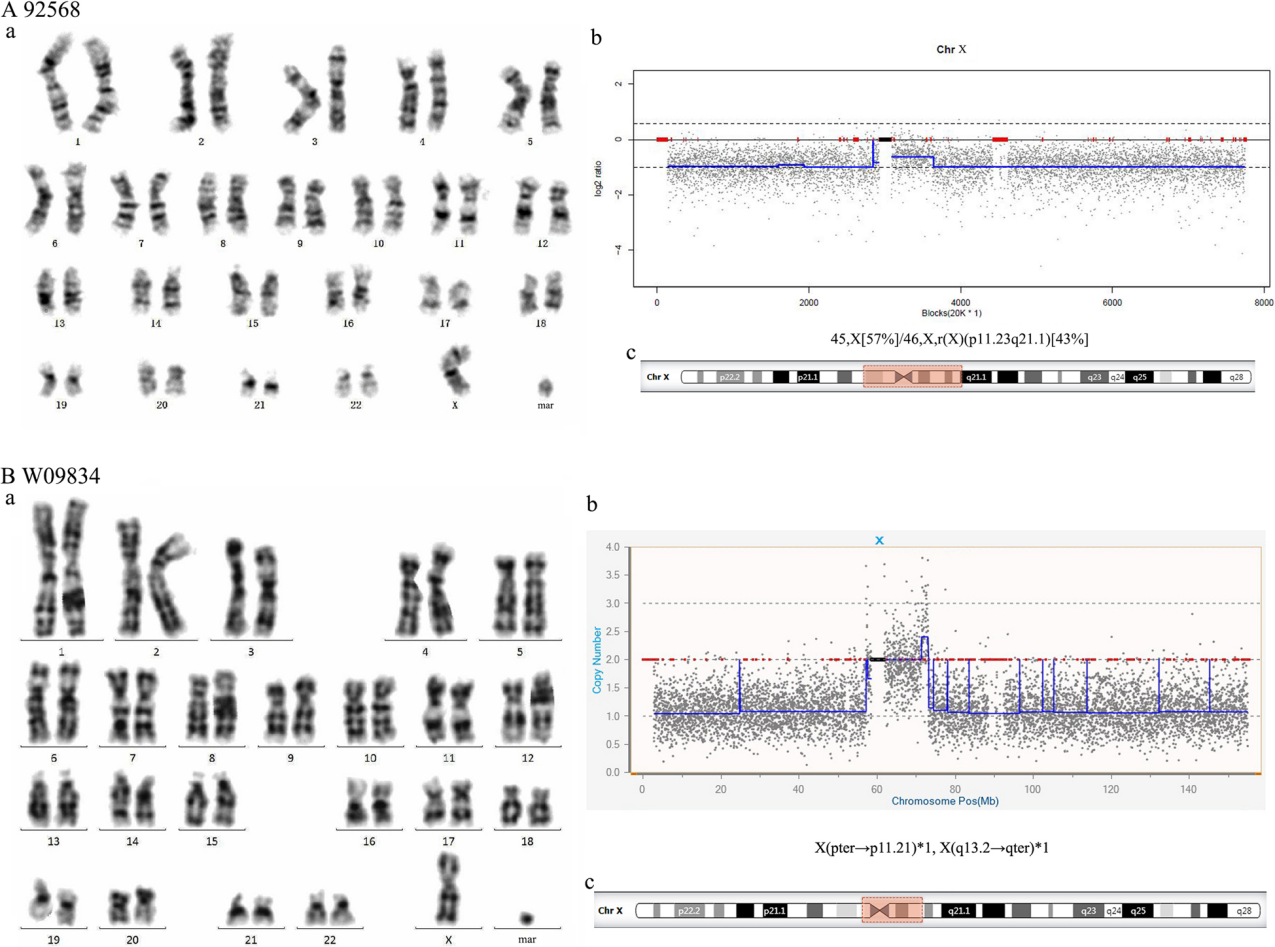
Cytogenetic and molecular results for patients carrying sSMCs derived from r(X) and min(X). The date of 92568 and W09834 were presented separately in (**A**) and (**B**). (**a**) The karyotypes were revealed by G-banding. (**b**) NGS detected the chromosome X. NGS results of W09834 were partial 45,X: X(pter→p11.21)×1, X(q13.2→qter)×1. It indicated that her sSMC was from X(p11.21→q13.1)(14.5 Mb). (**c**) The location of the sSMC on chromosome X is highlighted in red

The sSMCs of two patients were derived from chromosome X. These patients showed characteristics of Turner syndrome. NGS indicated that the sSMC of patient 92568, which was mosaic (45,X/46,X,+mar), might be from r(X) (Fig. 4A). The sSMC of patient W09834 was partial 45,X and composed of min(X) (Fig. 4B).

### sSMCs from chromosome 18

**Fig. 5:**
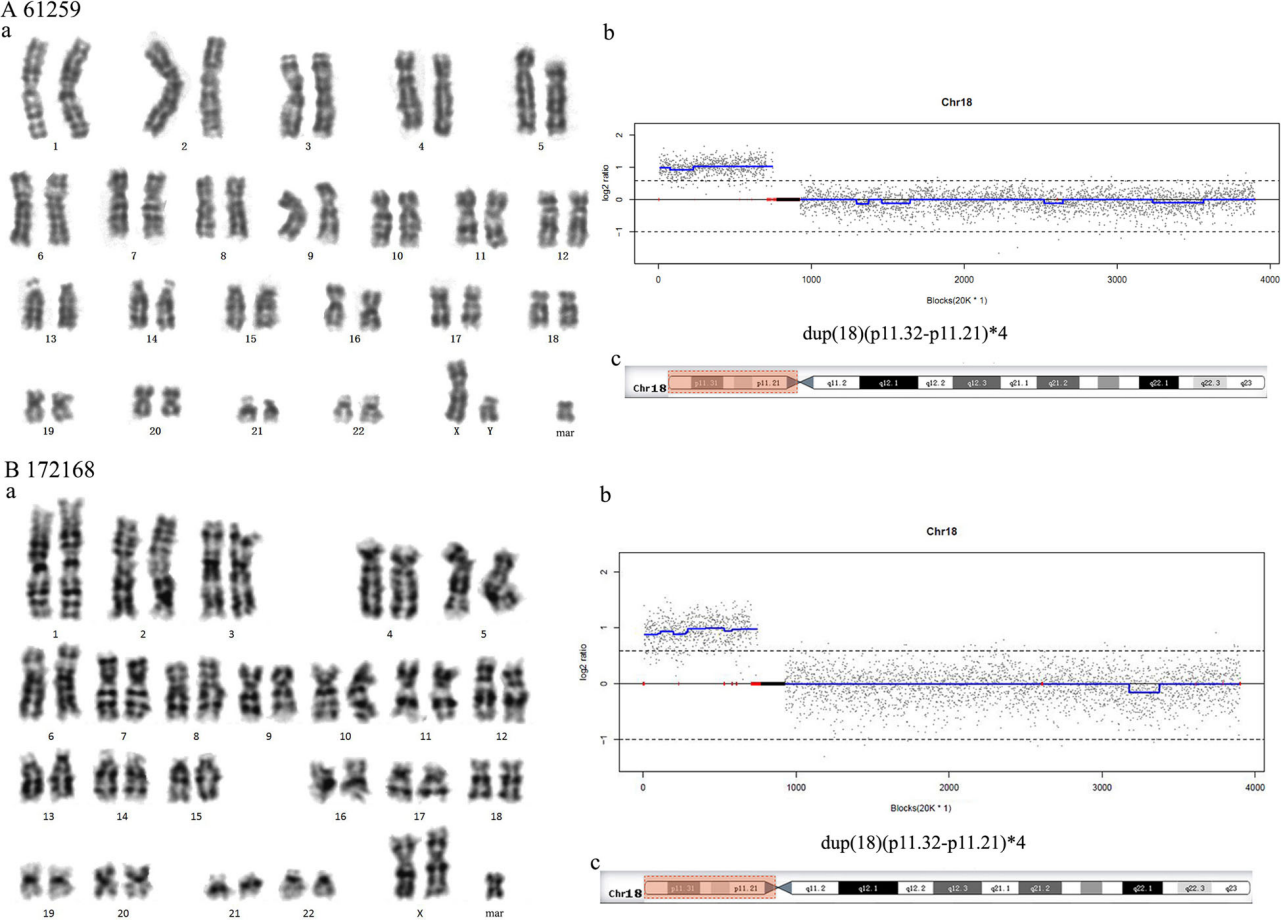
Cytogenetic and molecular results for patients carrying sSMCs derived from inv dul(18). The date of 61259 and 172168 were presented separately in (**A**) and (**B**). (**a**) The karyotypes were revealed by G-banding. (**b**) NGS determined duplications on chromosome 18. (**c**) The location of the sSMC on chromosome 18 is highlighted in red

The sSMCs of patient 61259 and fetus 172168 were derived from inv dul(18) (Fig. 5A, B). NGS showed that they had the genotype dup(18)(p11.32→p11.21)×4. It has been reported that the clinical symptoms are likely isochromosome 18p [i(18p)] syndromes or tetrasomy 18p syndrome, which feature neonatal feeding problems, hypoevolutism, and high risk of infections [5, 6].

### sSMCs from other chromosomes

**Fig. 6:**
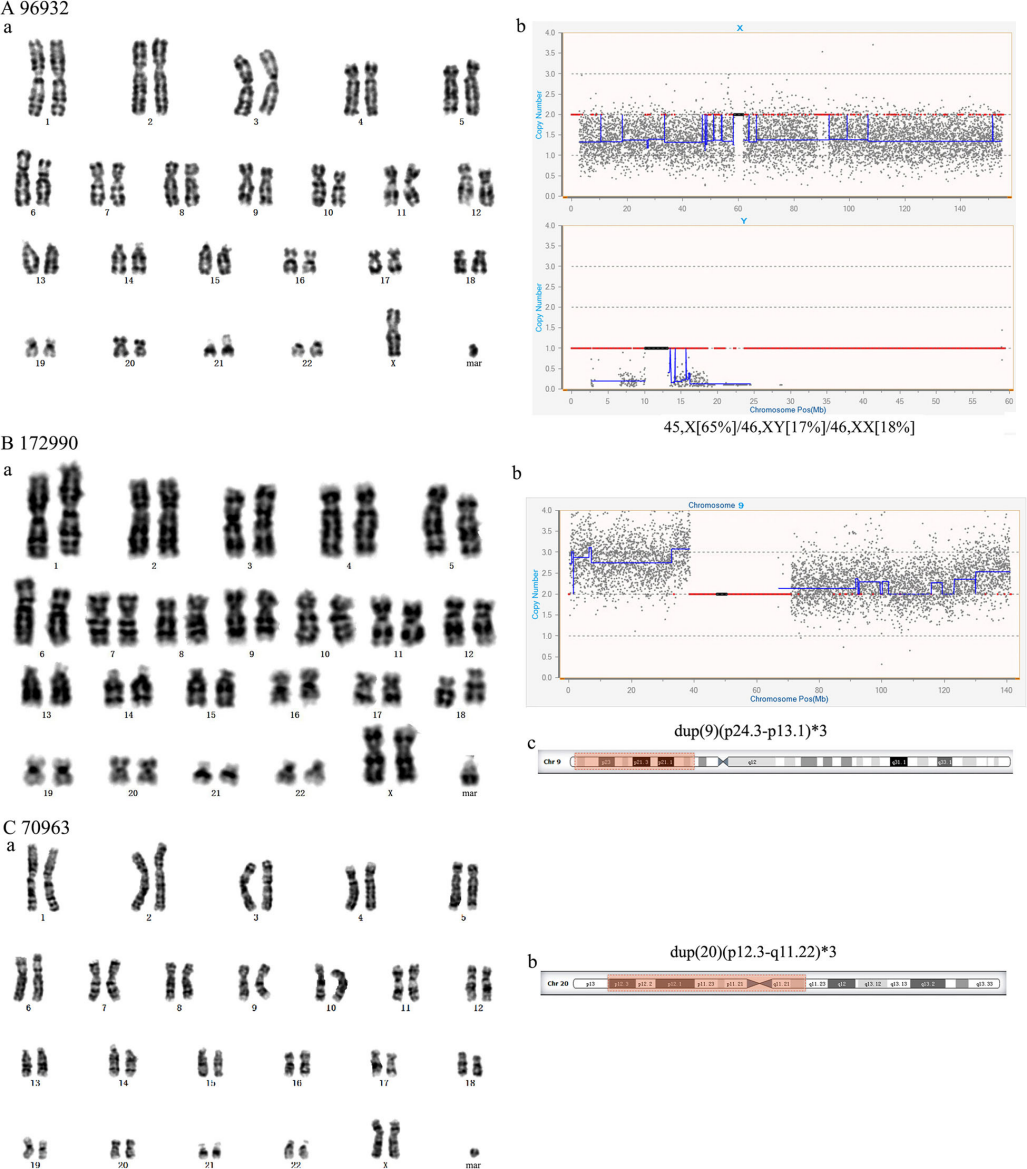
(**A**) Cytogenetic and molecular results for patient 96932. (**a**) The karyotype was revealed by G-banding. (**b**) NGS detected chromosome X and Y. (**B**) Cytogenetic and molecular results for patient 172990. (**a**) The karyotype was revealed by G-banding. (**b**) NGS identified duplication on chromosome 9. (**c**) The location of the sSMC on chromosome 9 is highlighted in red. (**C**) Cytogenetic result for patient 70963. (**a**) The karyotype was revealed by G-banding. (**b**) The location of the sSMC on chromosome 20 is highlighted in red

**Fig. 7:**
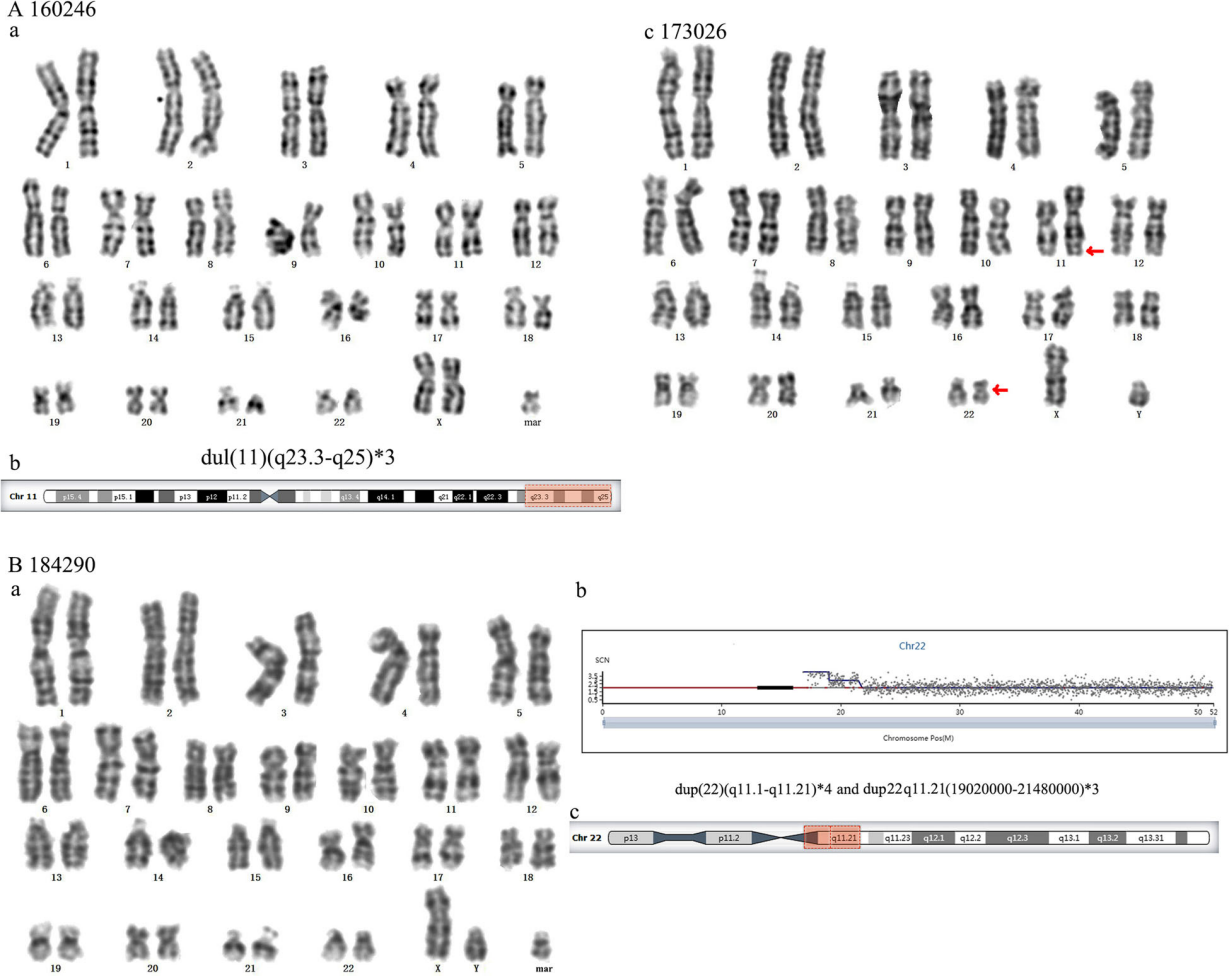
(**A**) Cytogenetic results for patient 160246. (**a**) The karyotype of patient 160246 was revealed by G-banding. (**b**) The location of the sSMC on chromosome 11 is highlighted in red. (**c**) The karyotype of 173026 carrying the same balanced translocation as his mother was revealed by G-banding. (**B**) Cytogenetic and molecular results for patient 184290. (**a**) The karyotype was revealed by G-banding. (**b**) NGS identified duplications on chromosome 22. (**c**) The location of the sSMC on chromosome 22 is highlighted in red

NGS showed that patient 96932 had a complex sSMC that might be derived from min(X) and min(Y) (Fig. 6A). This patient displayed similar characteristics to Turner syndrome. The sSMC of fetus 172990 was derived from min(9) (Fig. 6B). The sSMC of patient 70963, who showed compound features of partial trisomy 20p and 20q11.22 duplication syndrome with pygmyism and asitia, was derived from min(20) (Fig. 6C). The sSMC of fetus 160246 was derived from min(11) (Fig. 7A-a, b). When her mother got pregnant again, the fetus carried the same balanced translocation (Fig. 7A-c). The sSMC of fetus 184290 was derived from inv. dup(22) (Fig. 7B).

### sSMCs of unknown chromosomal origin

**Fig. 8:**
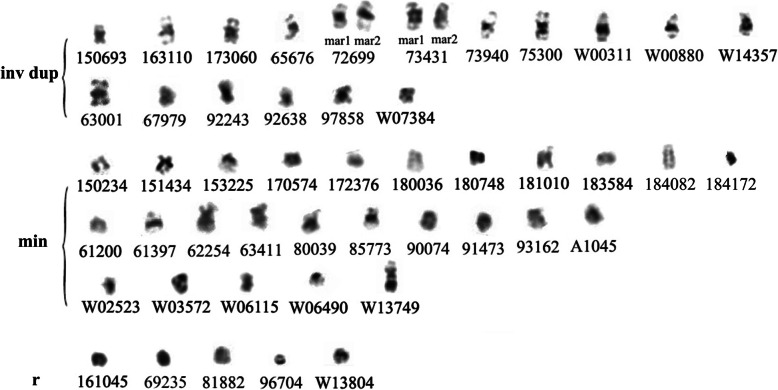
From karyotyping, unidentified sSMCs were classified into three groups. I: inverted duplicated chromosomes; II: minute chromosomes; III: ring chromosomes

Although several techniques were used to identify the origin of the different sSMCs, 48 patients could not be diagnosed (Table 2). Amniotic fluid samples containing sSMCs were submitted for STR analysis, and only seven sSMCs were identified. From karyotyping, these unidentified sSMCs were classified into three groups (Fig. 8). Group I sSMCs consisted of inverted duplicated chromosomes. Those in group II were likely minute chromosomes, while those in group III looked like ring chromosomes.

## Discussion

In this study, we identified the origins of 27 sSMCs, of which, eight sSMCs are being reported for the first time (Table 1). Of the 27 defined sSMC origins, 12 were derived from the Y chromosome and two from the X chromosome. The infertile patients showed azoospermia, and their original Y sSMCs were detected. Azoospermia factor (AZF), which is located on the long arm of Y (Yq11.23), regulates spermatogenesis [7]. These patients had deletions of AZF-a region (the Sertoli cell-only syndrome), AZF-b region (sperm-maturation-arrest syndrome), or all AZF regions resulting in azoospermia. Thus, artificial insemination with donor sperm or adoption was suggested for clinical management. The pediatric patients carrying sSMCs from min(Y) or chromosome X or complex sSMCs from min(X) and min(Y) had similar characteristics to Turner syndrome; however, they had different phenotypes depending on their sSMC origins. The short arm of X harbors the short stature-homeobox gene (*SHOX* on Xp22.33) and lymphogenic gene (forkhead box *P3*, *FOXP3* on Xp11.23), which are associated with stature and immunodeficiency or polyendocrinopathy [8]. Patient W09834 with min(X) had a loss of *FOXP3* and an immunological problem. A similar sSMC derived from r(X)(::p11.21→q13.1::) was reported in craniofrontonasal syndrome (CFNS) [9]. The methyl-CpG binding protein-2 gene (*MECP2* on Xq28) is located on the long arm of X. This gene correlates with RETT syndrome and the premature ovarian failure gene *POF* (*POF1*: Xq21→qter, *POF2*: Xq13.3→Xq21.1) [10]. As the min(X) from patient W09834 (:p11.2→q13.2:) and r(X) from patient 92568 (::p11.23→q21.1::) did not contain *SHOX* and *MECP2*, both patients had growth retardation and a high risk of RETT syndrome. As they had the part of *POFs*, so being attention to ovarian function. Patient 96932 had a complex sSMC from min(X) and min(Y), resulting in a high risk of type II germ cell tumors [11, 12]. All the pediatric patients were recommended for individualized treatment according to their genotype-related phenotypes.

Our sSMC patients with the 47,XN,+mar karyotype typically had special duplication syndrome, and six sSMCs were identified from inv dul(15). The region 15(q11.2→q13.3) is a known hot breakpoint. This region harbors the *GABAAR* genes, the paternal gene *SNRPN*, and the maternal gene *UBE3A,* which regulate central neural system development and function [13]. It was rare that two neocentric sSMCs derived from inv dup(18) had the same duplication fragment. There may be a hot breakpoint located at 18(p11.21). In region 18p, approximately 67 genes can contribute to the phenotypes, including *AFG3L2*, *MC2R*, and *TGIF1*, which are associated with developmental disorders [5, 6]. So, when taking care of patient 61259, pay attention to artificial feeding, avoiding infections, and evaluating affected organs and systems. The region of 20(p12.3→q11.22) comprises more than 2 hundred genes. Duplication of *JAG1, BTBD3,* and *FLRT3*, or *ASXL1* induces Alagille syndrome, neurological dysfunction or chromatin remodeling [14, 15]. Patient 70963 with the genotype min(20)(:p12.3→q11.22:) showed moderate symptoms due to 60% mosaic.

The identification of sSMCs is vital in prenatal diagnosis. Of the 75 sSMC cases from this study, 23 were from fetuses with intrauterine growth retardation or abnormal ultrasonic structure, and seven fetal sSMC cases were found to have Y, 18, 9, 11, or 22 chromosomal origins. However, most sSMCs failed to define the original chromosome. Three fetal sSMCs from the Y chromosome needed careful evaluation. If the sSMCs correlated with androgyneity or AZF deletion, it was better to complete the pregnancy. However, if a fetus had an inv dup(18) genotype, termination of the pregnancy was suggested because of the i(18p) syndromes. Fetus 172990 had a duplicated region 9(p24.3→p13.1) that correlated with 9p duplication syndrome, which contains a potential autism spectrum disorder (ASD) and a normal IQ individual region [16, 17]. The sSMC of fetus 160246 was de novo and arose from a maternal balanced translocation t(11;22)(q23;q12), leading to three copies of 11(q23.3→q25). The sSMC derived from the inv dup(22) chromosome was also de novo. The fetus carrying this sSMC had similar regions to the 22q11.2 duplication syndrome (22DupS), which usually produces birth defects, such as congenital heart disease, hearing loss, hypophrenia, or high risk of psychosis (including autism) [18, 19]. A similar sSMC arising from inv dup(22)(q11.1 ~ 11.2) was reported with mild clinical signs [20].

Most sSMCs in fetuses are de novo, but a few are inherited from their parents. Thus, prenatal diagnosis and genetic counseling are critical. In our department, parents are asked to fill out a form to collect genetic information. Amniotic fluid is then submitted for both karyotyping and STR analysis. If an sSMC is diagnosed, further testing (e.g., NGS) is suggested, and the karyotypes of the parents are requested. If the parents are sSMC or translocation carriers, the fetus should take further testing. Preimplantation genetic screening (PGS) and preimplantation genetic diagnosis (PGD) would help reduce the chances of miscarriage.

Although several sequencing-related techniques were used in our study, there were still 30 sSMCs for which pathogenic information could not be generated. It is possible that the sequencing primers did not cover the sSMC regions in the MLPA or STR (AZF) methods. Also, inverted duplicated chromosomes (acrocentric chromosomes), isochromosomes, or minute chromosomes (centromere-nearby regions) might not have been detected by NGS due to the highly repeated sequences at the centromere regions, which will be improved in read depth, inducing read pair, split pair, or assembly-based analysis of NGS. Thus, a set of efficient techniques should be developed for further sSMC identification.

## Conclusions

In summary, the sSMCs of the study patients were different in origin, size, replication times, affected genes, and mosaicism levels. Thus, their clinical manifestations varied. This study detailed the comprehensive characterization of 27 sSMCs. Eight of these sSMCs are being reported here for the first time, which provides additional information for sSMC research. The identification of sSMCs could reveal genotype-phenotype correlations and integrate genomic data into clinical care.

## Methods

### Patients’ collection

This research investigated 74,266 patients’ specimens in our department from 2015 to 2018, including 50,794 peripheral bloods from adults, 6,350 peripheral bloods from pediatrics, 14,759 amniotic fluids, and 2,363 cord bloods. 75 sSMC carriers were diagnosed by karyotyping (Tables 1 and 2), containing 52 live births, and 23 fetuses. Some of them took further detection (e.g., NGS, MLPA, or STR). Then we identified the molecular component of 27 sSMC cases. They were compared with the information in http://cs-tl.de/DB/CA/sSMC/0-Start.html. These retrospective studies were approved by the ethical commission of the Shengjing Hospital of China Medical University (NO.2019PS423K).

### Chromosome karyotyping

Patients’ peripheral blood and amniotic fluid samples were cultured, harvested, and stained with Giemsa (G-banded) (at the resolution of approximately 300–400 bands) following the standard protocols. Then scanned in Lieca Cyto Vision (German) and analyzed according to the ISCN 2013.

### STR and AZF detection

In our department first-generation sequencing (FGS) (3730 DNA Analyzer, Singapore) was used to detect STR on five chromosomes (13, 18, 21, X, and Y in amniotic fluid), AZF(Yq11.2) and SRY(Yp11.31) of Y (in azoospermia adult). DNA was extracted by kit (BioBase, Chengdu, China) in Auto-Pure32A (ALL SHENG, Hangzhou, China), mixed with sequencing primers and Tag DNA Polymerase (Transgen, Beijing, China), and then did PCR (S1000 Thermal Cycler) and sequenced.

### MLPA

MLPA was performed in FGS (3730 DNA Analyzer, Singapore) by the protocol of “SALSA® MLPA® P245 Microdeletion Syndromes-1” kit (MRC Holland, Amsterdam, the Netherlands). The preparation of DNA samples was same as STR. MLPA could suggest 23 kinds of deletion or duplication syndrome. Sequencing primers were illustrated in protocol, including one of Xp21.1, three of Xq28, three of 15q11.2 (one UBE3A probe and two SNRPN probes), and one (Y-fragment S0135-L16766) for the Y chromosome. MLPA data were presented with ratio.

### Next generation-sequencing

NGS was performed in accordance with the protocols of a commercial NGS sequencing kit (Berry Genomics, Hangzhou, China). DNA samples were prepared by the extract kit (Axygen, MA, USA), purified and enriched library, then sequenced in the Illumina NextSeq CN500 (Berry Genomics, Hangzhou, China). Sequencing data were analyzed with Software VI (Berry Genomics, Hangzhou, China) in h19 database, blasted and searched information of disease in DGV, DECIPHER, OMIM, UCSC and Pubmed. Data were presented with log2 ratio or copy numbers (SCN).

## Data Availability

The datasets used or analyzed during the current study are available from the corresponding author on reasonable request.

## Abbreviations

sSMC: Small supernumerary marker chromosomes
NGS: Next-generation sequencing
MLPA: Multiplex ligation-dependent probe amplification
STR: Short tandem repeats
AZF: Azoospermia factor
FGS: First-generation sequencing
NT: Nuchal translucency
NIPT: Non-invasive prenatal testing
MGS: Mixed gonadal dysgenesis
ART: Assisted reproductive technology
PGS: Preimplantation genetic screening
PGD: Preimplantation genetic diagnosis
LCRs: Low copy repeats
